# The Role of Nuclear Phosphoinositides in the p53-MDM2 Nexus

**DOI:** 10.3390/cells14151126

**Published:** 2025-07-22

**Authors:** Jeong Hyo Lee, Muhammad Khalil Salah, Xiangqin Chen, Nickolas Vladimir Kucherenko, Vincent L. Cryns, Richard A. Anderson

**Affiliations:** 1 University of Wisconsin Carbone Cancer Center, School of Medicine and Public Health, University of Wisconsin-Madison, Madison, WI 53705, USA; jlee2259@wisc.edu (J.H.L.); msalah@wisc.edu (M.K.S.); wilson.chen@wisc.edu (X.C.); kucherenko@wisc.edu (N.V.K.); 2 Department of Medicine, School of Medicine and Public Health, University of Wisconsin-Madison, Madison, WI 53705, USA

**Keywords:** phosphoinositide, p53, AKT, MDM2, small heat shock protein, nucleus

## Abstract

Recent insights into the p53-MDM2 nexus have advanced deeper understanding of their regulation and potent impact on cancer heterogeneity. The roles of nuclear phosphoinositide (PIP_n_s) in modulating this pathway are emerging as a key mechanism. Here, we dissect the molecular mechanisms by which nuclear PIP_n_s stabilize p53 through the recruitment of small heat shock proteins (sHSPs), activate the nuclear phosphatidylinositol 3-kinase (PI3K)-AKT signaling cascade, and modulate MDM2 function to regulate the p53-MDM2 interaction. We propose potential mechanisms by which nuclear PIP_n_s coordinate signaling with nuclear p53, AKT, and MDM2. Ultimately, we highlight that nuclear PIP_n_s serve as a ‘third messenger’ within the p53-MDM2 axis, expanding the current framework of non-canonical nuclear signaling in cancer biology.

## 1. Introduction

In nuclei, there is a full phosphoinositide signaling cascade that includes all of the phosphoinositide kinases and phosphatases. These enzymes are spatially located within the nucleus in regions that are separate from known membranes [1]. The spatially organization has been controversial because of the lack of membrane structures that hold the phosphatidylinositol lipids that localize with the phosphoinositide metabolizing enzymes. This suggests that phosphoinositide lipids are in a novel compartment [2]. Recently, it has been shown that phosphoinositides are linked to multiple nuclear proteins including p53, STAR-PAP, NRF2, and MDM2 in complexes that are indistinguishable from a posttranslational modification [3,4,5,6,7]. This suggests a novel organization of the phosphoinositide lipids in cells. Here, we will discuss the implications for the p53 and MDM2 nexus.

Tumor suppressor TP53 (p53) is a nuclear transcription factor that orchestrates cellular responses to stress by promoting cell-cycle arrest or apoptosis. It is the most frequently mutated gene across all cancer types, with mutation rates ranging from 38–50% in solid tumors [8,9]. Approximately 80% of theses alterations are missense mutations located within the central DNA-binding domain, with hotspot mutations at residues R175, G245, R248, R249, R273, and R282, which impair its transcriptional activity toward pro-apoptotic genes [10]. Mutant p53 not only loses its tumor-suppressive function, but also acquires oncogenic properties, referred to as gain-of-function mutations, that enable cancer cell survival under various stress condition, including hypoxia, oxidative stress, genotoxic stress, and endoplasmic reticulum stress [11].

**Figure 1:**
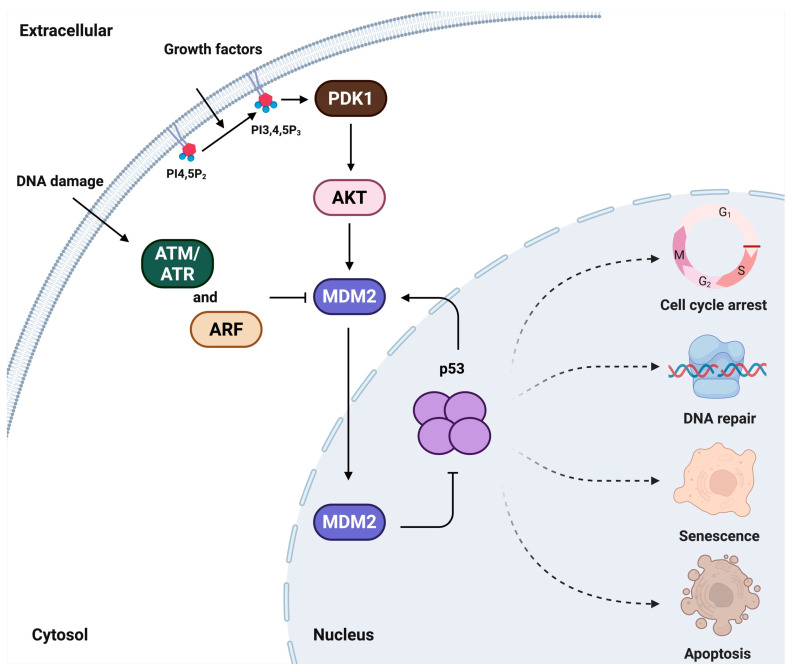
A historical model of p53-MDM2 nexus regulatory pathways. In cellular signaling, extracellular agonists act as first messengers by binding to membrane receptors, initiating pathways that promote either cell growth or DNA-damage response. In both contexts, PIP_n_s serve as second messengers that propagate the signal to elicit a cellular response. Under growth stimulation, activated growth factor receptors trigger the generation of PIP_3_ that activates the PI3K-AKT pathway, leading to MDM2 phosphorylation and its nuclear translocation, where it suppresses p53. Conversely, under DNA damage, kinases such as ATM/ATR or ARF inhibit MDM2, thereby stabilizing and protecting p53.

In contrast, MDM2 is a nuclear E3 ubiquitin ligase whose expression is transcriptionally induced by p53. MDM2 functions as a negative regulator of p53 by promoting its ubiquitination and subsequent degradation, thereby attenuating p53-mediated responses such as cell-cycle arrest, DNA repair, senescence, and apoptosis, particularly under conditions of AKT activation and genotoxic stress [12,13,14,15] (Figure 1). Clinically, MDM2 amplification, defined by increased gene copy number and elevated protein level, occurs in approximately 3.5% of cancer patients. Among this subgroup, 24.9% exhibit co-occurring alternations in the p53 pathway, and 25.4% indicate changes in the PI3K pathway. These findings suggest that MDM2 overexpression can concurrently disrupt both p53 function and PI3K signaling in the nuclei, contributing to tumor progression through multiple mechanisms [16].

The PI3K signaling cascade is dependent on phosphatidylinositol (PI) and the phosphorylated isomers phosphatidylinositol 4-phosphate (PI4P), phosphatidylinositol 4,5-bisphosphate (PI4,5P_2_), that ultimately generate phosphatidylinositol 3,4,5-bisphosphate (PI3,4,5P_3_), which regulates a broad array of cellular functions. While traditionally characterized as membrane components, emerging studies demonstrate that class I phosphatidylinositol transfer proteins (PITPs) transport PI into the nucleus, where it serves as a substrate for PI4K and PI3K enzymatic activity [17]. Consequently, phosphorylated PIP_n_s are linked to p53 and recruit AKT, 3-phosphoinositide-dependent kinase-1 (PDK1), and mammalian target of rapamycin complex 2 (mTORC2), and activate nuclear AKT to promote cell survival, invasion, and DNA repair [4]. Given the emerging evidence that PIP_n_s are linked to both p53 and MDM2, this commentary will critically address how nuclear PIP_n_s serves as a regulatory mechanism within the p53-MDM2 nexus contributing to oncogenic signaling and enhancing the adaptive advantages of cancer cells [18,19,20].

## 2. p53

Recent studies have revealed a mechanism by which nuclear PIP_n_s signaling is initiated through a direct linkage of PI4,5P_2_ to p53. Choi et al. demonstrated that one of the downstream lipid products, PI4,5P_2_, directly influences p53 stability [3]. They indicated that PI4,5P_2_ binds to a polybasic region within the C-terminal domain of p53, promoting its stabilization. This effect is not solely due to lipid association, but also requires the recruitment of sHSPs, including HSP27 and αB-crystallin. These chaperones associate with the p53–PI4,5P_2_ complex in a PI4,5P_2_-dependent manner and act as molecular stabilizers, preserving p53 protein integrity. These findings are consistent with earlier studies identifying sHSPs as critical regulators of p53 under stress conditions and reinforce the role of nuclear PI4,5P_2_ in scaffolding stable protein complexes in the nucleus [18,19].

Building on this nuclear PIP_n_ pathway, Carrillo et al. demonstrated that, under genotoxic stress, PITPα and PITPβ deliver PI, which becomes linked directly to p53 in the nucleus and supplies the PI that is complexed to the PITPs. This PI is phosphorylated by phosphatidylinositol 4-kinase II α (PI4KIIα) to generate PI4P, forming a p53–PI4P complex [17]. This complex is then sequentially converted into PI4,5P_2_ by type I phosphatidylinositol phosphate kinase α (PIPKIα), and subsequently to PI3,4,5P_3_ by inositol polyphosphate multi-kinase (IPMK) [3,4]. Through this phosphorylation cascade, p53 acts as a scaffold that organizes lipid kinases and downstream effectors that are regulated by the linked PIP_n_s. These discoveries establish p53 not only as a transcriptional regulator, but also as a platform coordinating lipid-mediated stress signaling in the nucleus.

Beyond this stabilization pathway, nuclear PIP_n_s also coordinate signaling pathways that are scaffolded on p53. This was reported by Chen et al., who discovered that under genotoxic stress, PI4,5P_2_ linked with p53 is phosphorylated by the PI 3-kinase IPMK to generate a p53-PI3,4,5P_3_ complex [4]. This complex functions as a non-canonical signaling platform that recruits the key PI3,4,5P_3_ effectors PDK1, mTORC2, and AKT. Their recruitment results in nuclear-localized activation of a PI3,4,5P_3_-dependent kinase assembly including AKT, which in turn phosphorylates downstream targets including Forkhead Box O (FOXO) transcription factors, DNA-dependent protein kinase (DNA-PK), and p300. Through these downstream signals, AKT facilitates DNA repair and enhances cell survival in response to genotoxic stress, establishing a direct mechanistic link between nuclear PIP_n_s signaling and stress-adaptive responses.

These findings underscore the role of nuclear PIP_n_s as dynamic regulators of p53, not only by stabilizing it through sHSPs recruitment, but also by assembling the stress-adaptive signaling pathway via nuclear AKT activation. The discovery that p53 scaffolds a nuclear AKT activation complex through PIP_n_s linkage marks a significant advance in understanding of how non-canonical PIP_n_s signaling integrates with stress-response pathways. Moreover, the regulation of p53 by nuclear PIP_n_s appears to establish a critical balance between promoting cell survival and permitting apoptosis. The activation of nuclear AKT via the p53-PI3,4,5P_3_ complex is positioned to support survival by facilitating DNA repair and inhibiting pro-apoptotic transcriptional activity driven by FOXOs [4]. Of note, when p53 is mutated, this results in a p53-PI3,4,5P_3_-AKT complex that is always active, shifting the balance toward proliferation. This suggests an underlying mechanism for mutant p53 gain of function oncogenic activity.

The broader significance of these findings is emphasized by clear evidence that nuclear PIP_n_s signaling extends beyond p53, influencing diverse nuclear processes such as transcriptional regulation, chromatin remodeling, and DNA replication [20,21,22]. In this context, nuclear PI4,5P_2_ and PI3,4,5P_3_ act not merely as secondary messengers, but as regulatory components essential for preserving genomic integrity. While studies by Carrillo, Choi, and Chen have laid a fundamental framework for understanding how PIP_n_s regulate p53, several key questions remain. It is still unclear how nuclear stress signals selectively activate PIPKIα and IPMK to modulate p53′s PIP_n_s association state, or whether p53 mutations that impair lipid or sHSPs binding contribute to tumorigenesis independently of its transcriptional activity.

## 3. MDM2

Recent work by Lee et al. discovered that PIP_n_s are linked to the E3 ubiquitin ligase MDM2 [7]. MDM2 is a key regulator of p53, controlling its ubiquitination and degradation via the proteasome [14]. The key advances in this article include: (i) genotoxic stress enhances the interaction of PIPKIα and its product PI4,5P_2_ with MDM2, thereby recruiting sHSPs, and (ii) PI4,5P_2_ determines the selective recruitment of distinct sHSPs to MDM2, ultimately influencing the fate of the p53-MDM2 nexus. These results support an emerging regulatory mechanism involving both binding and linkage of nuclear PI4,5P_2_ and MDM2, further discussion is required to understand the full implications of these findings.

PIP_n_s that are tightly linked to MDM2 and p53 in an association indistinguishable from posttranslational modification. MDM2 and p53 also bind PIP_n_s [3,4,7,17] and the data indicate that the linked PIP_n_s also serve to regulate their activities by intramolecular and likely by intermolecular binding. For p53, PI4,5P_2_ binds to the C-terminal domain, a key region that regulates many p53 functions [3,23]. These findings support a model in which nuclear PIP_n_s function beyond conventional second messengers. Instead, they act as ‘third messengers’, forming stable linkage with target proteins (PIPylation) and regulating their stability and activity through direct interaction.

In addition, the study presents a model for how PI4,5P_2_ modulates the p53-MDM2 nexus. Since both p53 and MDM2 are linked to and are associated with nuclear PIP_n_s, it has been difficult to dissect their individual regulatory pathways [24]. This study identifies sHSPs, ATP-independent molecular chaperones, as key determinants of specificity [25]. While nuclear PI4,5P_2_ recruits both HSP27 and αB-crystallin to p53 to enhance its stabilization [24], sHSPs play as active rather than passive chaperones to selectively regulate the p53-MDM2 axis.

Under stress conditions, PI4,5P_2_ functions as a molecular on-off switch that determine which sHSPs associate with MDM2. PI4,5P_2_ weakens the interaction between MDM2 and HSP27, thereby loosening the p53-MDM2 complex. In contrast, it enhances the interaction between MDM2 and αB-crystallin, stabilizing MDM2 and promoting tighter binding to p53. These differential associations also influence MDM2 ubiquitination activity. The in vitro ubiquitination assay indicated that αB-crystallin reduced ubiquitination activity, whereas both HSP27 and PI4,5P_2_ increased it. Notably, adding PI4,5P_2_ to this cell-free autoubiquitination assay did not alter the impacts mediated by HSP27 or αB-crystallin, indicating that sHSPs exert a more dominant regulatory influence over MDM2 ubiquitination activity than PI4,5P_2_ alone. Subsequent cellular experiments have examined how PI4,5P_2_ levels regulate MDM2 ubiquitination. The knockdown of PIPKIα led to decreased PI4,5P_2_ levels, which correlated with increased total ubiquitination and MDM2 ubiquitination, but reduced p53 ubiquitination. This was accompanied by decreases in MDM2 protein levels, which was reduced by treatment with the proteasome inhibitor MG132. These opposing effects of sHSPs enable PI4,5P_2_ for sophisticated fine-tuning of the complicated p53-MDM2 nexus.

Given that direct binding between p53 and MDM2 is essential for regulating p53 stability [26,27], this PI4,5P_2_-sHSPs regulatory axis has significant biological implications. However, several open questions remain unresolved. For instance, MDM2 has clusters of positively charged amino acids—classically known as PIP_n_s-binding motifs. But it has not yet been confirmed whether these regions directly serve as the PI4,5P_2_ site to MDM2. Additionally, it remains unclear whether these regions are also involved in linkage, or if a distinct linkage site exists on MDM2. Clarifying this point would be crucial for fully understanding the molecular basis of the association. Furthermore, although stress was shown to promote enhanced PI4,5P_2_ and MDM2 linkage, the mechanisms through which nuclear PIP_n_s recognize cellular stress signal remains elusive. To answer these open questions, continuous research efforts are required.

## 4. Potential Targets of the p53-MDM2-PIP_n_ Nexus

Nuclear PIP_n_s act as signaling messengers that regulate both p53 and MDM2 through the selective recruitment of specific sHSPs. These PIP_n_s-s-HSPs-p53 or MDM2 complexes are positioned to modulate select targets by the recruitment of different interactors, for example sHSPs. These regulated assemblies may play a role in nuclear signaling pathway and stress response regulation.

### 4.1. Nuclear p53 and Its Targets

p53 regulates a wide range of targets to maintain genomic stability and suppress tumorigenesis. The first identified transcriptional target of p53 is CDKN1A (p21) [28]. p21 functions as an inhibitor of cyclin-dependent kinases (CDKs), leading to decreased levels of cell-cycle proteins and subsequent inhibition of retinoblastoma (RB) phosphorylation, thereby contributing to p53-mediated G1 phase arrest [29,30,31]. To promote apoptosis, p53 activates several pro-apoptotic targets including p53 upregulated modulator of apoptosis (PUMA), BCL2 associated X apoptosis regulator (Bax), and phorbol-12-myristatn-13-acetate-induced protein 1 (Noxa) [32,33,34]. Additionally, p53 regulates p16^INK4A^ (p16), which suppresses CDK4/6 activity, reduces RB phosphorylation, and inactivates E2F, leading to either cell-cycle arrest or senescence [35,36]. Beyond direct cell-cycle and apoptotic control, p53 exerts a significant influence on the tumor microenvironment: Wild-type p53 enhances anti-tumor immune reposes and indicates synergistic effects with cancer immunotherapy [37,38], whereas mutant p53 promotes a tumor-permissive microenvironment that supports cancer cell progression [39,40,41]. Obviously, MDM2 and AKT are also key downstream targets regulated by p53.

### 4.2. Nuclear AKT and Its Targets

Chen et al. presented compelling evidence that nuclear PIP_n_s stabilize p53, leading to the generation of the p53-PI3,4,5P_3_ complex that recruits AKT, PDK1, and mTORC2, which activates a non-canonical nuclear AKT signaling pathway [4]. Through this mechanism, nuclear PIP_n_s not only regulates AKT, but also could modulate upstream and downstream effectors of the AKT pathway. To be specific, PDK1 is a central kinase that activates AKT via phosphorylation and, directly, phosphorylates polo-like kinase 1 (PLK1), which promotes MYC phosphorylation and accumulation, thereby enhancing cancer cell proliferation [42,43]. In addition, PDK1 stabilizes hypoxia-inducible factor 1α (HIF1α) by inhibiting its ubiquitination, further contributing to tumor progression [44].

Beyond AKT activation, mTORC2 also phosphorylates a member of the protein kinase C (PKC) family, which plays key roles in promoting cancer cell invasion [45,46,47,48]. mTORC2 also phosphorylates serum- and glucocorticoid-induced kinase 1 (SGK1), which supports cancer cell survival [49,50]. AKT itself activates nuclear factor kappa-light-chain-enhancer of activated B cells (NF-κB) through phosphorylation, sustaining oncogenic signaling [51,52,53,54]. Furthermore, AKT phosphorylates pro-apoptotic proteins BAD and BAX, thereby inhibiting apoptosis and enhancing cell survival [55,56]. Intriguingly, AKT reinforces the p53-MDM2 negative feedback loop by promoting MDM2 stability in the nucleus, thereby contributing to the regulation of p53 levels in a manner analogous to cytoplasmic signaling [57]. For these pathways, the roles of the nuclear p53-AKT signalosome need to be further defined, but this complex is clearly spatially positioned as a major mediator of PDK1, mTORC2, and AKT in the nuclei.

## 5. p53-Independent Functions of MDM2

Although MDM2 is best known as a negative regulator of p53, it also targets several pathways independent of p53. For example, during Ras-ERK pathway activation, FOXO3a is phosphorylated and becomes a substrate for MDM2-mediated ubiquitination, which suppresses FOXO3a activity and promotes cell proliferation [58]. MDM2 also ubiquitinates E-cadherin, contributing to early tumor metastasis by promoting epithelia-to-mesenchymal transition (EMT); meanwhile, reduced E-cadherin disrupts cell adhesion and polarity, facilitating invasion and metastasis [59,60,61].

While p53 regulates cell-cycle arrest, MDM2 induces cell-cycle progression by enhancing the transcription of E2 promoter binding factor 1 (E2F1) and transcription factor DP1 (DP1), supporting S-phase entry and protecting p53-deficient cell from E2F-mediated apoptosis [62,63]. Additionally, MDM2 directly interacts with Nijmegen breakage syndrome 1 (Nbs1), suppressing DNA-damage response signaling and attenuating ATM activation [64]. MDM2 also ubiquitinates ribosomal protein L26 (RPL26), thereby inhibiting p53 translation, and insulin-like growth factor 1 receptor (IGF-1R), regulating proliferation independently of p53 [65,66,67,68]. In the context of epigenetic regulation, MDM2 promotes mono-ubiquitination of histone H2A and H2B, leading to transcriptional repression and gene silencing, which also ubiquitinates dihydrofolate reductase (DHFR), thus influencing folate metabolism [69,70]. How MDM2-PIP_n_ complexes participate in these processes remains to be defined.

## 6. Conclusions

**Figure 2:**
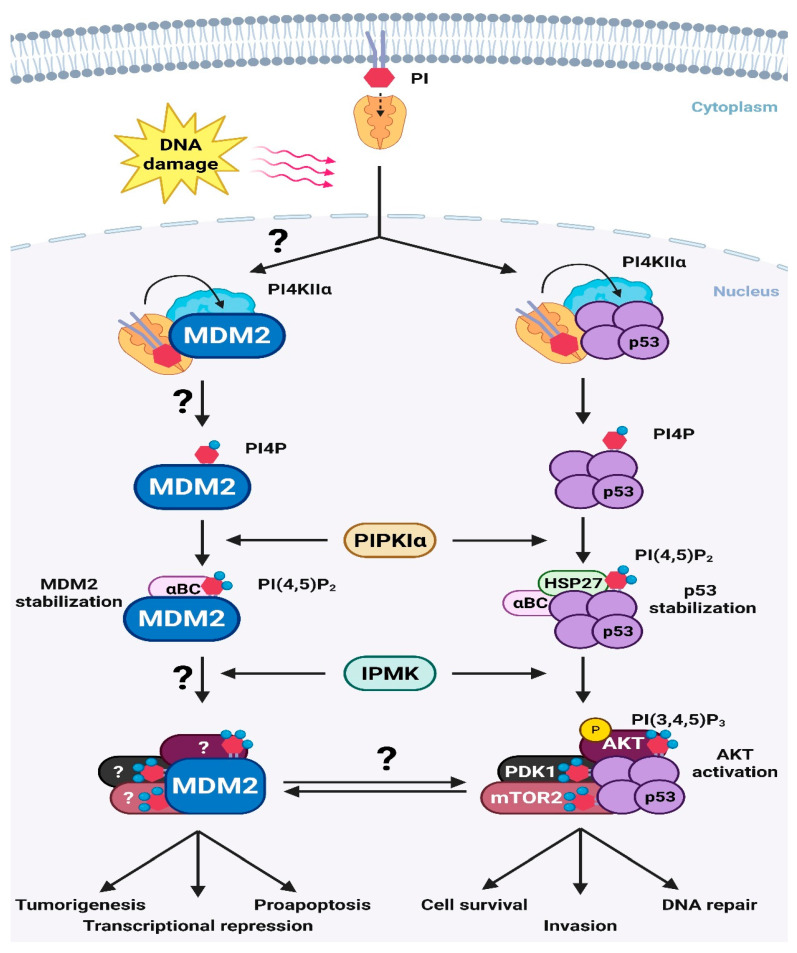
A model of non-canonical ‘third messengers’ regulating the p53-MDM2 nexus. PITPs transport PI from the membrane into the nucleus, initiating the PIPylation process. PI4KIIα, PIPKIα, and IPMK sequentially convert PI into various PIP_n_ species. These nuclear PIP_n_s modulate the p53-MDM2 nexus and, consequently, regulate several critical cancer-related biological processes. Question marks indicate pathways that have not yet been experimentally validated.

Nuclear PIP_n_s play a central role in regulating both p53 and MDM2. By recruiting specific sHSPs, nuclear PIP_n_s fine-tune the p53-MDM2 nexus, positioning this pathway to modulate a broad range of biological functions (Figure 2). This process begins with PITP, which initiates the PIPylation of p53 by transferring PI from membranes to p53 and possibly MDM2 and other targets. PI4KIIα subsequently coverts the p53-PI complex into a p53-PI4P complex, which is further interconverted by PIPKIα and IPMK into various PIP_n_ species. These modifications stabilize p53 and activate nuclear AKT signaling. Although the initiation of MDM2 PIPylation is not defined, it likely involves similar PI transfer and interconversion mechanisms as overserved for p53. Collectively, these findings support the model in which nuclear PIP_n_s function as a ‘third messenger’, stably linked to p53-MDM2 nexus and regulates their function and stability. Given that both p53 and MDM2 govern critical cellular processes, particularly those related to tumor suppression and progression, nuclear PIP_n_s are likely to influence a range of cancer-related biological functions by modulating this regulatory nexus.

Additionally, the discoveries of nuclear PIP_n_ cancer-related targets obviously underscores the cancer treatment potential [3,4,5,7,71]. As stated above, PIPKIα and its product PI4,5P_2_ are coupled with p53 to stabilize mutant p53 by recruiting sHSPs and contribute mutant p53 oncogenicity [3]. Subsequently, PI3,4,5P_3_-p53 complexes generated by IPMK promote nuclear AKT pathways and demonstrate tumorigenic phenotypes [4]. On the other hand, nuclear PIP_n_s also regulate oncoprotein MDM2 stability and functions that target p53 [7]. While the therapeutic landscape remains largely unexplored, these observations indicate that interfering with PIP_n_ in the p53-MDM2 nexus or their upstream kinases may provide a strategy to selectively disrupt oncogenic signaling pathways in cancer.

Despite these advances and promising potential of non-canonical PIP_n_s signaling, several important questions remain unanswered. Although additional PIPylated proteins continue to be identified, further studies are needed to elucidate how these modifications influence cellular and pathological functions, both in vitro and in vivo. In conclusion, recent discoveries have revealed unpredicted roles for nuclear PIP_n_s in the regulation of the p53-MDM2 nexus. This non-canonical PIP_n_s regulation of p53-MDM2 nexus is likely to contribute to a wide array of diseases, especially cancer.

## Data Availability

No new data were created or analyzed in this study.
